# Silk Fibroin Peptides Promote Extracellular Matrix Homeostasis in Photoaging via Modulation of the ITGB1/FAK-TGF-β/Smad Signaling Axis

**DOI:** 10.3390/molecules31162820

**Published:** 2026-08-13

**Authors:** Siyuan He, Yongqiu Yan, Feifei Xiong, Wenwen Diao, Fuhuai Jia, Xiaodong Yan, Jing Wang

**Affiliations:** 1 Key Laboratory of Synthetic and Biological Colloids, Ministry of Education, School of Chemical and Material Engineering, Jiangnan University, Wuxi 214122, China; 6240606005@stu.jiangnan.edu.cn; 2 Zhejiang Daldrem Biotechnology Co., Ltd., Ningbo 315153‌, China; yanyongqiu@hotmail.com (Y.Y.); wen17mail@163.com (W.D.); aaafast@sina.com (F.J.)

**Keywords:** silk fibroin peptide, photoaging, extracellular matrix homeostasis, integrin β1 (ITGB1), human dermal fibroblasts

## Abstract

Excessive ultraviolet A (UVA) irradiation disrupts extracellular matrix (ECM) homeostasis in skin photoaging by impairing the balance between synthesis and degradation, yet whether silk fibroin peptide (SF), a small bioactive peptide from *Bombyx mori*, can restore this balance through mechanotransduction pathways remains unknown. Herein, we demonstrate that SF dose-dependently rescues human dermal fibroblasts (HDFs) from UVA-induced oxidative stress, senescence, and ECM disintegration. Notably, SF not only suppresses reactive oxygen species (ROS) and restores activities of antioxidant enzymes, but is also associated with the recovery of the ITGB1-FAK mechanotransduction axis, as evidenced by restored fibronectin levels and increased focal adhesion kinase (FAK) phosphorylation, whereas integrin β1 (ITGB1) expression itself was not significantly altered. This mechanosensory recovery is accompanied by restoration of downstream transforming growth factor-β (TGF-β)/Smad signaling, upregulation of *COL1A1*, *COL3A1* and *ELN* transcription, and simultaneous suppression of *MMP1*, *MMP3* and *MMP9*. Unlike conventional antioxidants or exogenous collagen supplements that merely counteract oxidative damage or provide structural substitutes, SF may facilitate recovery of the disrupted cell–matrix interface potentially through modulation of integrin-mediated mechanochemical signal conversion, which may contribute to ECM homeostasis restoration. Collectively, SF promotes mechanotransduction, offering a potential paradigm for anti-aging strategies that target ECM homeostasis through integrin signaling.

## 1. Introduction

Ultraviolet (UV) radiation is the primary exogenous factor inducing skin photoaging [1]. Specifically, ultraviolet A (UVA) with a wavelength of 320–400 nm can penetrate to the dermis, accounting for approximately 80% of extrinsic facial skin aging [2]. UVA radiation induces excessive generation of reactive oxygen species (ROS) through activation of endogenous photosensitizers [3]. In addition to directly causing oxidative damage to lipids, proteins, and DNA, excessive ROS can also act as signaling molecules to trigger a cascade of degenerative changes in dermal connective tissue [4]. In this process, ROS upregulates the expression of matrix metalloproteinases, including *MMP1*, *MMP3*, and *MMP9*, thereby promoting the degradation of collagen and elastin in the extracellular matrix (ECM) [5]. Meanwhile, ROS inhibits the synthetic capacity of fibroblasts for ECM components such as collagen and elastin [6]. These lead to a dynamic imbalance between ECM synthesis and degradation, resulting in decreased ECM content and ultrastructural disorder, and further promoting the formation of clinical photoaging features such as skin laxity and wrinkles [7].

The process of photoaging involves not only the loss of ECM components but also the impairment of fibroblasts’ ability to sense and respond to the microenvironment [8]. Fibroblasts recognize the mechanical properties and biochemical signals of the ECM via integrin receptors on the cell surface, converting mechanical stimuli into intracellular signaling cascades [9]. Among integrin subunits, integrin β1 (ITGB1) is the most highly expressed one in fibroblasts, and the heterodimers formed by its combination with different α subunits are the primary receptors mediating cell-ECM adhesion [10]. Integrin receptors containing ITGB1 cluster at the cell membrane to form focal adhesions upon binding to ECM ligands, recruiting and activating focal adhesion kinase (FAK) [11]. Activated FAK serves as a key signal transduction node, regulating cell proliferation, migration, and ECM synthesis by mediating cytoskeletal remodeling and activating downstream PI3K-Akt and MAPK-ERK pathways [12]. Consistent with this mechanism, studies have shown that UVA irradiation downregulates ITGB1 expression and reduces FAK phosphorylation, thereby disrupting the mechanotransduction pathway and impairing the ability of fibroblasts to sense and respond to microenvironmental cues, ultimately leading to ECM homeostasis imbalance in photoaging. This mechanosensory deficit not only weakens cell-ECM adhesion but also attenuates TGF-β-driven ECM synthesis [13]. In this context, the transforming growth factor-β (TGF-β)/Smad signaling axis, as the core regulatory pathway for ECM synthesis, exhibits a close functional association with the ITGB1-FAK mechanosensing pathway. On one hand, integrin-mediated cytoskeletal contractile force is critical for activating latent TGF-β complexes stored in the ECM and releasing active TGF-β ligands. On the other hand, activated FAK can enhance the signal transduction efficiency of TGF-β receptors, promoting the phosphorylation and nuclear translocation of Smad2/3 [14], thereby upregulating the transcription of ECM genes such as *COL1A1*, *COL3A1*, and tropoelastin (*ELN*). Therefore, restoring the functional coupling of the ITGB1-FAK-TGF-β/Smad signaling axis is a promising strategy for repairing photoaging-induced ECM damage.

Natural bioactive peptides have attracted widespread attention in the field of skin repair due to their excellent biocompatibility and multiple biological activities [15]. Silk fibroin peptide (SF) is a low-molecular-weight bioactive peptide extracted from *Bombyx mori* silk fibroin [16]. It consists primarily of glycine (~40%), alanine (~30%), and serine (~10%), which is similar to that of collagen [17]. Previous studies demonstrated that SF possessed favorable transdermal absorption properties [18] and exerted protective effects on the extracellular matrix by scavenging reactive oxygen species and inhibiting matrix metalloproteinase activity [19]. However, current research has largely focused on the antioxidant [20] and ECM-degradation-inhibitory effects of SF, and whether it can regulate the ITGB1/FAK mechanotransduction pathway to restore TGF-β/Smad-driven collagen synthesis and reverse ECM homeostasis imbalance remains unknown. To address this gap, we hypothesize that SF may promote ECM homeostasis through combined antioxidant, anti-senescence, and ECM-protective effects, with the recovery of mechanotransduction potentially occurring as a consequence of ECM preservation rather than direct integrin activation. Using a UVA-induced photoaging model in human dermal fibroblasts [21], we observed that SF treatment was associated with recovery of the ITGB1/FAK-TGF-β/Smad signaling and may contribute to rebalancing ECM synthesis and degradation. These findings may offer a molecular rationale for developing ECM-homeostasis-targeted anti-aging interventions [10].

## 2. Results and Discussion

### 2.1. SF Attenuates UVA-Induced Oxidative Stress and Cellular Senescence in HDFs

Prior to mechanistic investigations, preliminary experiments were conducted to establish optimal experimental parameters. CCK-8 assay demonstrated that SF exerted no significant cytotoxicity toward HDFs across the tested concentration range of 6.25 to 1000 μg/mL (Figure S1), indicating a favorable safety profile for subsequent intervention studies. UVA dose–response analysis revealed that irradiation at 30 J/cm^2^ reduced cell viability to approximately 70 percent of the control level (Figure S2), a condition that elicited substantial cellular damage while preserving sufficient viable cells for pharmacological assessment [22]. Based on these findings, SF concentrations of 12.5, 50, and 100 μg/mL were selected as low, medium, and high doses, respectively, to evaluate dose-dependent effects.

**Figure 1:**
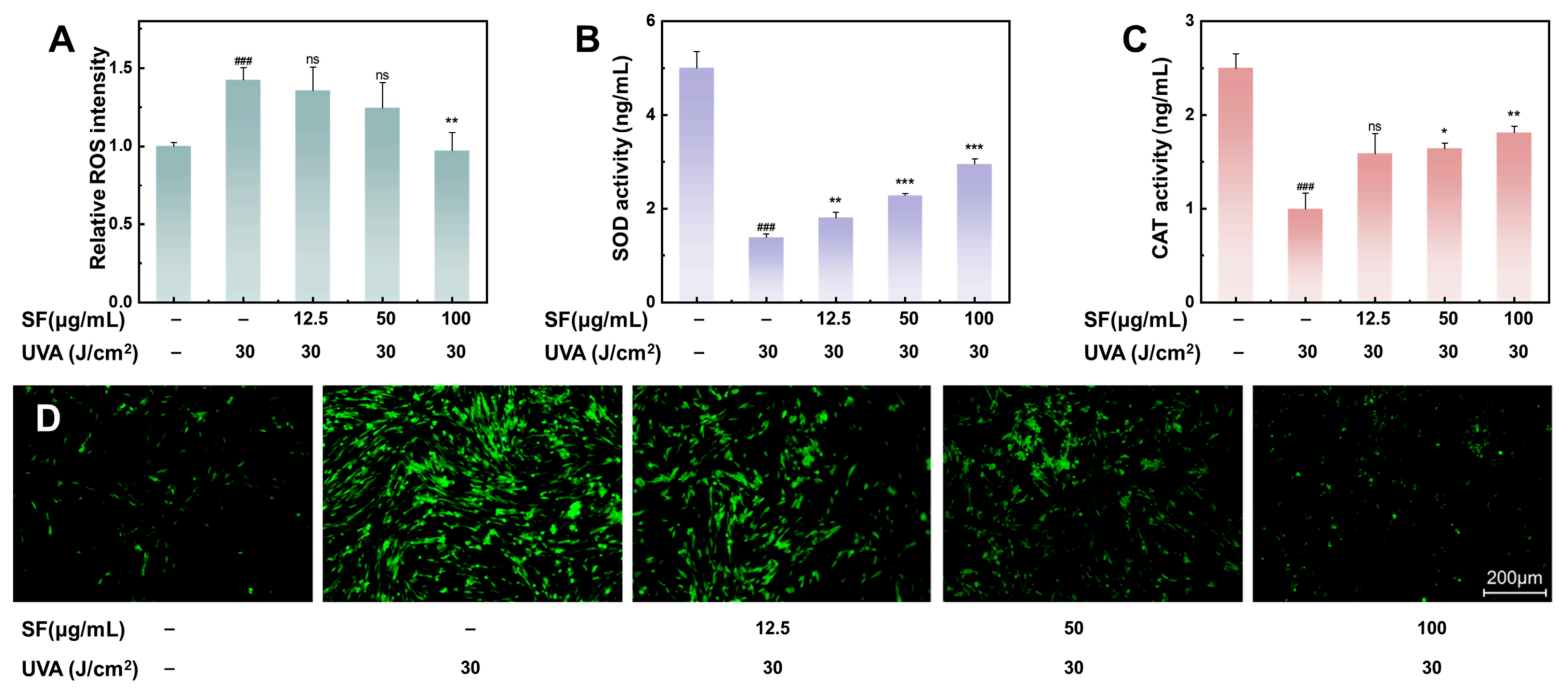
Effect of silk fibroin peptide (SF) on UVA-induced oxidative stress in human dermal fibroblasts (HDFs). (**A**) Intracellular reactive oxygen species (ROS) levels detected by DCFH-DA fluorescence probe. (**B**) Superoxide dismutase (SOD) activity. (**C**) Catalase (CAT) activity. (**D**) Representative DCFH DA fluorescence images. The scale bar is 200 μm. Data are presented as mean ± SD, *n* = 3. Statistical significance: ### *p* < 0.001 versus control group; * *p* < 0.05, ** *p* < 0.01, *** *p* < 0.001 versus UVA model group; ns, not significant.

**Figure 2:**
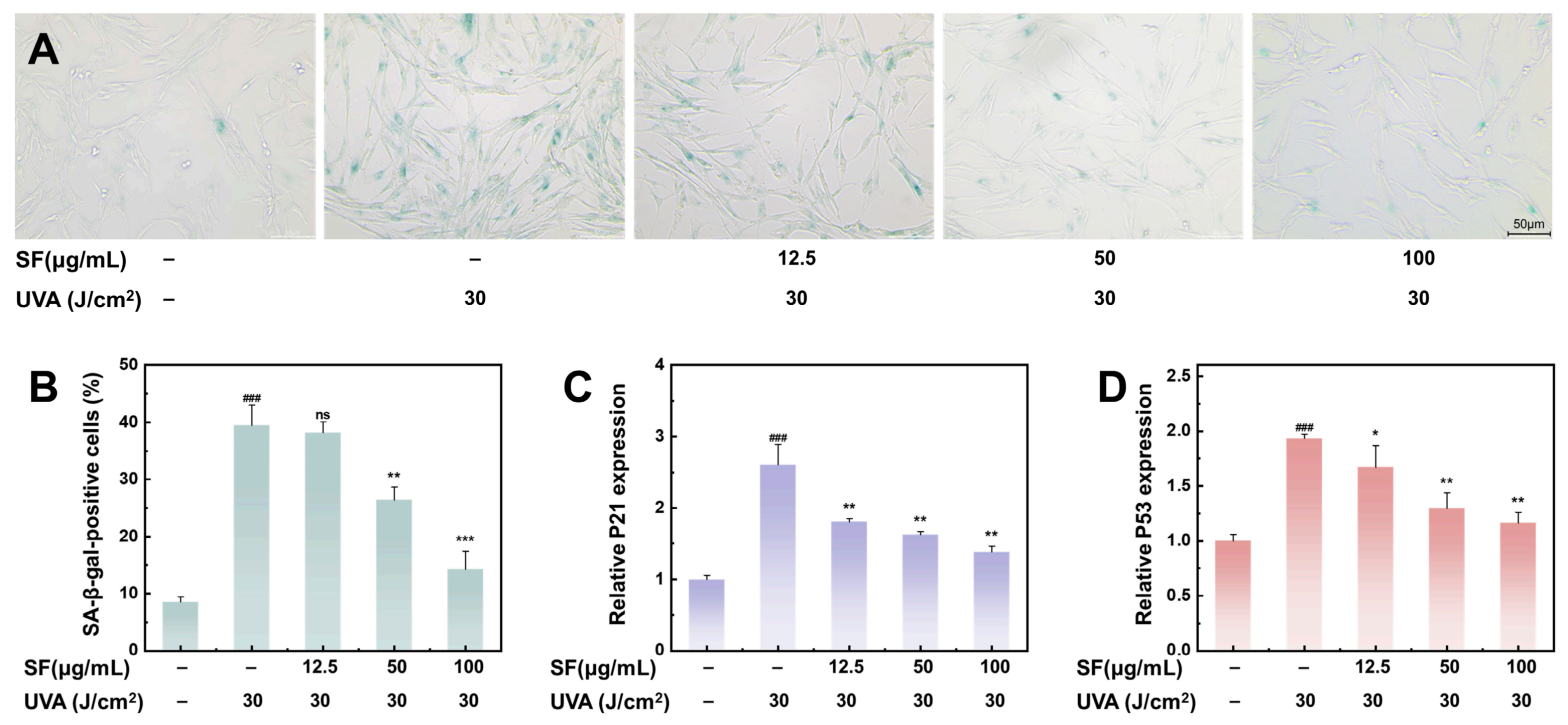
Effects of SF on UVA-induced HDF cell senescence and related signaling pathways. (**A**) SA-β-gal staining for cellular senescence. The scale bar is 50 μm. (**B**) Percentage of SA-β-gal-positive cells. (**C**) Relative mRNA expression of *P21*. (**D**) Relative mRNA expression of *P53.* Data are presented as mean ± SD, *n* = 3. Statistical significance: ### *p* < 0.001 versus control group; * *p* < 0.05, ** *p* < 0.01, *** *p* < 0.001 versus UVA model group; ns, not significant.

**Figure 3:**
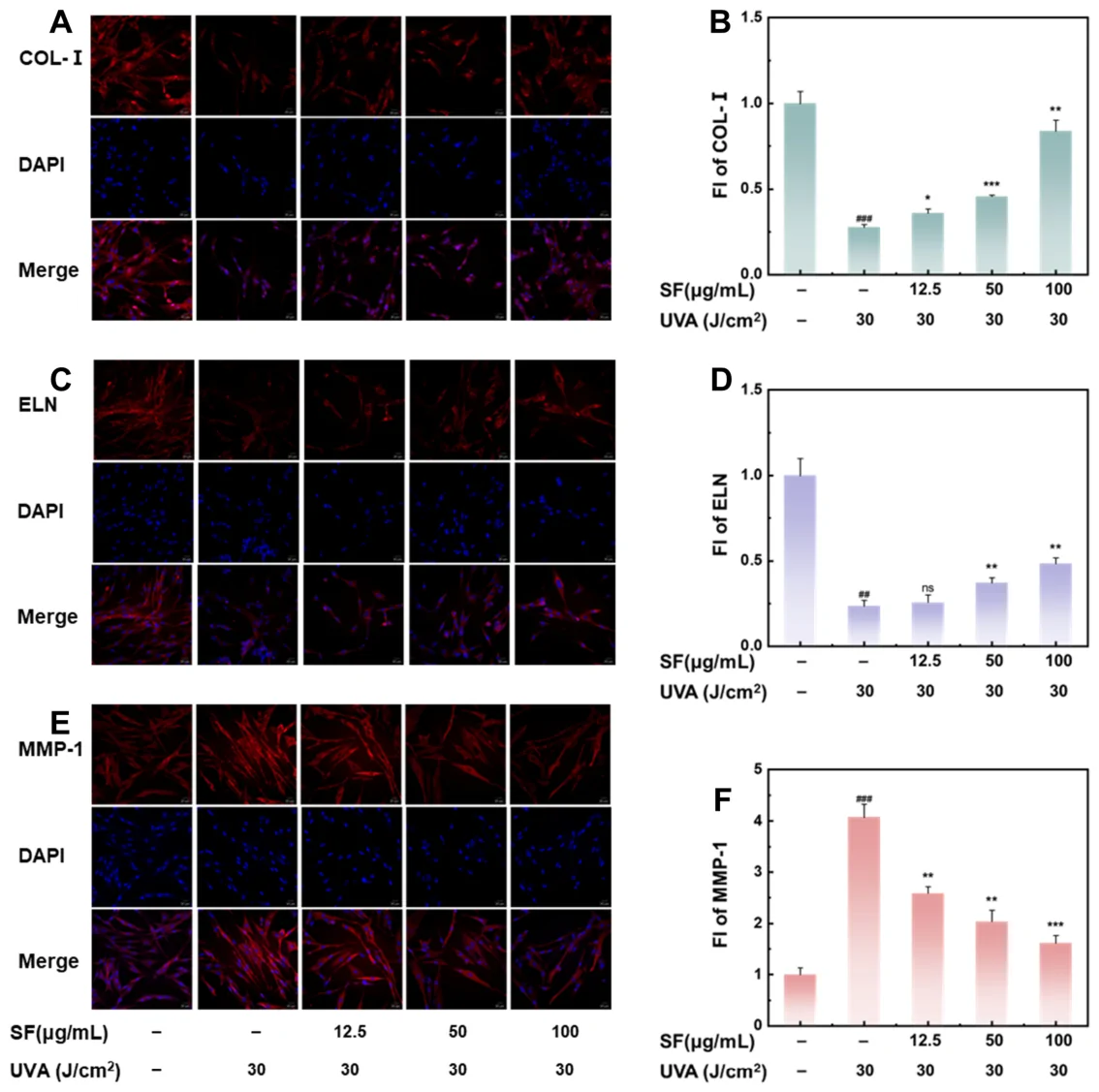
Immunofluorescence detection and quantitative analysis of the effects of SF on the expression of key ECM proteins in UVA-injured HDF cells. (**A**) Immunofluorescence staining of type I collagen (COL-I). (**B**) Relative fluorescence intensity of COL-I. (**C**) Immunofluorescence staining of elastin (ELN). (**D**) Relative fluorescence intensity of ELN. (**E**) Immunofluorescence staining of MMP-1. (**F**) Relative fluorescence intensity of MMP-1. Data are presented as mean ± SD, *n* = 3. Statistical significance: ## *p* < 0.01, ### *p* < 0.001 versus control group; * *p* < 0.05, ** *p* < 0.01, *** *p* < 0.001 versus UVA model group; ns, not significant. The scale bar is 200 μm for all immunofluorescence images.

**Figure 4:**
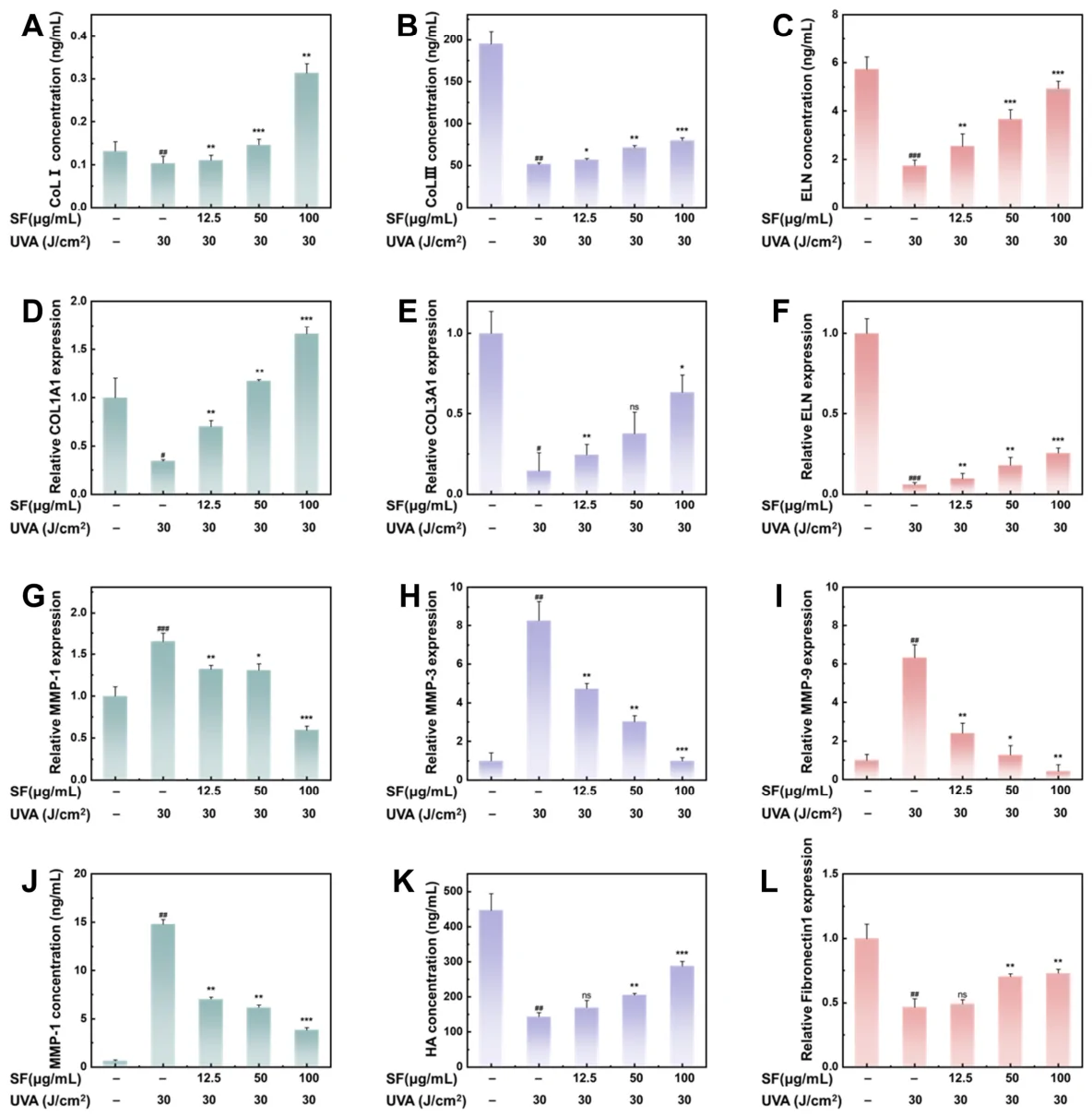
Effect of SF on ECM anabolic and catabolic balance in UVA-irradiated HDFs. (**A**–**C**) ELISA analysis of COL-I, COL-III, and ELN protein levels. (**D**–**F**) qPCR analysis of *COL1A1*, *COL3A1*, and *ELN* mRNA expression. (**G**–**I**) qPCR analysis of *MMP1, MMP3,* and *MMP9* mRNA expression. (**J**,**K**) ELISA analysis of MMP-1 and HA protein levels. (**L**) qPCR analysis of *FN1* mRNA expression. Data are presented as mean ± SD, *n* = 3. Statistical significance: # *p* < 0.05, ## *p* < 0.01, ### *p* < 0.001 versus control group; * *p* < 0.05, ** *p* < 0.01, *** *p* < 0.001 versus UVA model group; ns, not significant.

UVA irradiation markedly elevated intracellular ROS levels and concomitantly suppressed endogenous antioxidant enzyme activities in HDFs [23]. As shown in Figure 1A, ROS levels in the UVA-exposed model group were significantly higher than those in the control group (*p* < 0.001). Following SF intervention, ROS fluorescence intensity decreased in a dose-dependent manner, with the high concentration group being significantly lower than the model group (*p* < 0.01). Consistently, intracellular superoxide dismutase (SOD) and catalase (CAT) activities were markedly reduced following UVA irradiation relative to the control group. Both differences were statistically significant (*p* < 0.001 for each, as shown in Figure 1B for SOD and Figure 1C for CAT). SF treatment dose-dependently restored both enzyme activities, with the high concentration group approaching control levels (both *p* < 0.01). Fluorescence micrographs revealed densely distributed and intense green fluorescent signals within cells of the model group, whereas SF intervention gradually attenuated the fluorescence intensity, with the high concentration group resembling the normal control (Figure 1D). These findings indicate that SF effectively alleviates UVA-induced oxidative stress damage in HDFs, as reflected by reduced ROS levels and restored endogenous antioxidant enzyme activities. This mitigation of oxidative stress likely contributes to the subsequent suppression of cellular senescence and ECM-degrading factors, a well-established relationship [24], as also observed in our experiments (Figure 2, Figure 3 and Figure 4).

SA-β-gal staining revealed that the percentage of SA-β-gal-positive cells in the UVA-exposed model group was significantly elevated relative to the control group (*p* < 0.001). This result is shown in Figure 2A,B. Following SF intervention, the proportion of positive cells decreased in a dose-dependent manner, with the high concentration group being significantly lower than the model group (Figure 2C,D, *p* < 0.01). Further analysis of gene expression showed that UVA irradiation markedly upregulated the expression of both *p21* and *p53* relative to the control group (*p* < 0.001 for each). SF treatment dose-dependently downregulated the expression levels of both genes, with the high-concentration group being significantly lower than the model group (both *p* < 0.01). These findings indicate that SF alleviates UVA-induced senescence phenotypes in HDFs and is associated with suppression of the activation of the *p53-p21* signaling axis, a well-established mediator of cellular senescence [25].

### 2.2. SF Restores ECM Structural Integrity in UVA-Damaged HDFs

Immunofluorescence staining revealed that UVA irradiation markedly attenuated extracellular Col I (Figure 3A,B) and elastin fluorescence (Figure 3C,D) signals relative to the control group, with the pericellular collagen network exhibiting sparse and fragmented morphological alterations. It is well established that UVA exposure leads to degradation of Col I and elastin in the ECM [26]. Concurrently, MMP-1 fluorescence signals were markedly intensified and diffusely distributed throughout the extracellular matrix (Figure 3E,F). Such spatial redistribution suggests that UVA not only downregulates the expression of structural proteins but also induces matrix metalloproteinases that degrade collagen and other extracellular matrix proteins, thereby disrupting fiber assembly and anchoring within the ECM and resulting in a loss of physical continuity of the dermal scaffold [27]. Following SF intervention, Col I and elastin fluorescence intensities recovered in a dose-dependent manner, with the high concentration group restoring a dense extracellular fibrillar network and MMP-1 signals being significantly retracted (all *p* < 0.01). These findings suggest that the protective effects of SF on the ECM may extend beyond the restoration of protein abundance to also include maintenance of proper assembly and spatial localization of collagen and elastic fibers within the extracellular space, which may be important for preserving the mechanical integrity of the dermis.

### 2.3. SF Rebalances ECM Synthesis and Degradation in Photoaged HDFs

The core pathological hallmark of photoaging is not simply depletion of individual proteins, but rather a net imbalance between ECM anabolic and catabolic pathways [28]. ELISA and qPCR data corroborated this bidirectional regulatory mechanism at the molecular level (Figure 4). On the synthetic side, UVA irradiation significantly downregulated the protein concentrations of COL-I, COL-III, and elastin (Figure 4A–C), with concomitant suppression of *COL1A1* and *COL3A1* transcription as well as *ELN* transcription (Figure 4D–F), suggesting blockade of the fibroblast collagen synthesis program (all *p* < 0.001). Following SF intervention, both protein and mRNA levels recovered in a dose-dependent manner, with *COL1A1* expression in the high-concentration group approaching control levels (*p* < 0.01). Given that type I collagen, encoded by *COL1A1*, constitutes over 80% of total dermal collagen, its transcriptional activation is important for maintaining dermal density and tensile strength; the restorative effect of SF at this locus is therefore of notable anti-aging relevance.

On the degradative side, UVA irradiation markedly upregulated *MMP1*, *MMP3*, and *MMP9* gene expression (Figure 4G–I) (all *p* < 0.001) alongside MMP-1 protein concentration (Figure 4J). Notably, MMP-1 is the key enzyme responsible for cleaving the triple-helical domains of type I and III collagen, and can lead to ECM scaffold disintegration when excessively activated [29]. SF treatment reduced MMP-1 protein and *MMP3* gene expression to levels significantly below those of the model group (both *p* < 0.01), and dose-dependently downregulated *MMP1* and *MMP9* transcription.

Concurrently, ECM microenvironmental components were also ameliorated: the UVA-induced decline in hyaluronic acid (HA) concentration (Figure 4K) and fibronectin (*FN1*) mRNA expression (Figure 4L) was partially restored following SF intervention, suggesting recovery of matrix hydration capacity and cell–matrix adhesion functionality. Collectively, SF does not merely supplement exogenous collagen, but may contribute to ECM metabolic homeostasis through a dual mechanism of anabolic activation and catabolic suppression. This mechanism involves upregulation of *COL1A1*, *COL3A1*, and *ELN* transcription to promote structural protein synthesis, downregulation of *MMP1*, *MMP3*, and *MMP9* to limit proteolytic degradation, and coordinated restoration of the HA–fibronectin microenvironment. This homeostatic rebalancing likely serves as a key mechanism through which SF exerts dermal-level anti-aging effects, and offers a foundation for further elucidating its upstream signal transduction mechanisms.

### 2.4. SF Is Associated with Recovery of ITGB1/FAK Mechanotransduction

**Figure 5:**
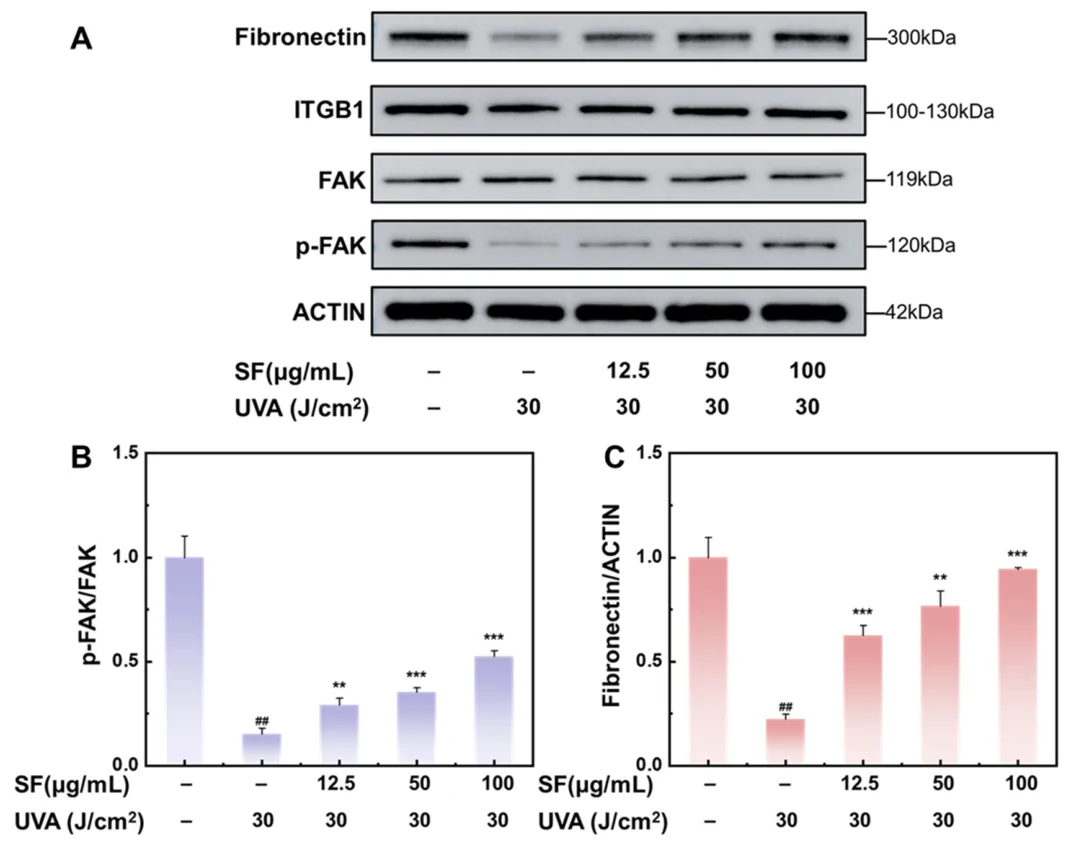
Regulatory effects of SF on key proteins in the ITGB1/FAK signaling pathway in UVA-injured HDF cells. (**A**) Western blot analysis of Fibronectin, ITGB1, FAK, and p-FAK protein expression. (**B**) Quantitative analysis of p-FAK/FAK phosphorylation levels. (**C**) Relative expression level of Fibronectin/ACTIN. Data are presented as mean ± SD, *n* = 3. Statistical significance: ## *p* < 0.01 versus control group; ** p < 0.01, *** p < 0.001 versus UVA model group.

Western blot analysis (Figure 5A) revealed that fibronectin, ITGB1 protein expression, and p-FAK phosphorylation levels in HDFs were significantly reduced following UVA irradiation relative to the control group (*p* < 0.01). This suggests disruption of cell–matrix mechanical connectivity and impairment of focal adhesion signaling platforms [30]. Quantitative analysis (Figure 5B) showed that the p-FAK/FAK ratio recovered in a dose-dependent manner following SF intervention, with the high concentration group reaching levels significantly higher than those of the model group (*p* < 0.001). Concurrently, the Fibronectin/ACTIN ratio was also restored in a dose-dependent manner (*p* < 0.001), as shown in Figure 5C. Notably, while UVA reduced ITGB1 expression, SF treatment did not significantly restore ITGB1 protein levels, suggesting that the recovery of integrin-mediated signaling may occur primarily through preservation of ECM ligand availability (e.g., fibronectin) rather than through direct upregulation of the integrin receptor itself. These findings suggest that SF treatment is associated with restoration of ECM ligand availability (e.g., fibronectin), which may indirectly support ITGB1/FAK-mediated mechanosensory recovery and improve cellular perception of the ECM microenvironment. [31]. Recovery of the ITGB1/FAK pathway may support downstream TGF-β/Smad signaling activation [10].

### 2.5. SF Is Associated with Recovery of TGF-β/Smad Signaling and Collagen Synthesis

**Figure 6:**
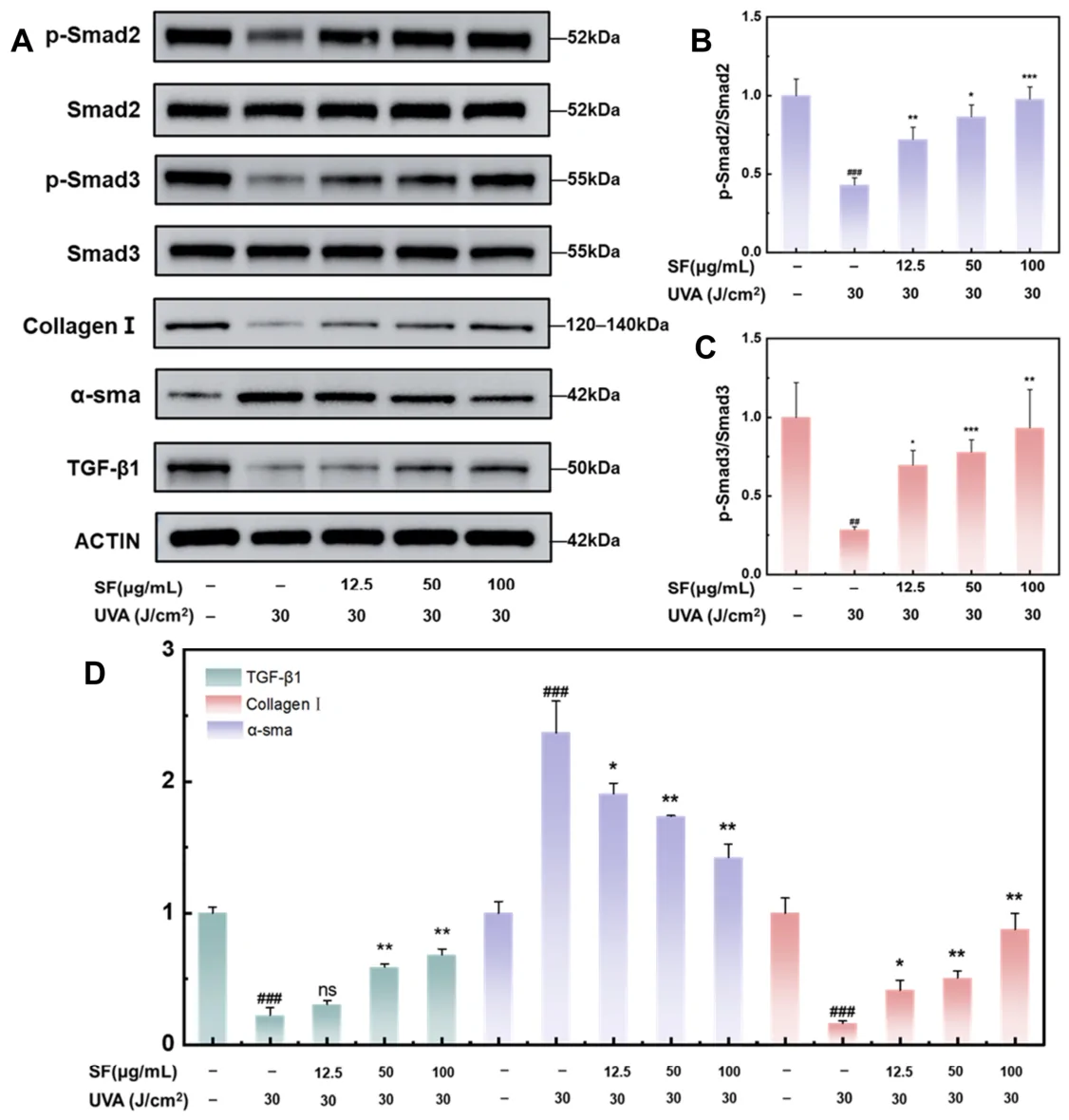
Regulatory effects of SF on the TGF-β/Smad signaling pathway and fibrosis-related proteins in UVA-injured HDF cells. (**A**) Western blot analysis of TGF-β1, p-Smad2, Smad2, p-Smad3, Smad3, Collagen I, and α-SMA protein expression. (**B**) Quantitative analysis of p-Smad2/Smad2 phosphorylation levels. (**C**) Quantitative analysis of p-Smad3/Smad3 phosphorylation level. (**D**) Relative expression levels of TGF-β1, Collagen I, and α-SMA. Data are presented as mean ± SD, *n* = 3. Statistical significance: *## p* < 0.01, *### p* < 0.001 versus control group; * p < 0.05, ** p < 0.01, *** p < 0.001 versus UVA model group; ns, not significant.

Following SF treatment, the recovered fibronectin availability may indirectly support FAK activity, which has been reported to amplify TGF-β signal transduction through downstream cascades including PI3K–Akt [32], thereby promoting Smad2/3 phosphorylation and nuclear translocation to transcriptionally upregulate ECM genes such as *COL1A1* and *ELN*, ultimately contributing to the restoration of ECM homeostasis. To examine whether this mechanotransduction-to-synthesis cascade was functionally restored by SF, we next assessed TGF-β/Smad signaling and its downstream effectors. Western blot analysis (Figure 6A) revealed that TGF-β1 protein expression and Smad2/3 phosphorylation levels in HDFs were significantly reduced following UVA irradiation relative to the control group (*p* < 0.001). Following SF intervention, TGF-β1 expression recovered in a dose-dependent manner, with the p-Smad2/Smad2 and p-Smad3/Smad3 ratios being synchronously restored (Figure 6B,C). The high concentration group approached control levels (both *p* < 0.01). Downstream effector protein detection revealed that Collagen I and α-SMA expression was significantly downregulated following UVA irradiation and recovered in a dose-dependent manner upon SF treatment (Figure 6D, *p* < 0.001). Collectively, these findings suggest that SF treatment is associated with the recovery of ECM synthetic signaling, potentially involving the ITGB1/FAK mechanosensing pathway and downstream TGF-β/Smad activity, which may contribute to ECM homeostasis restoration.

**Figure 7:**
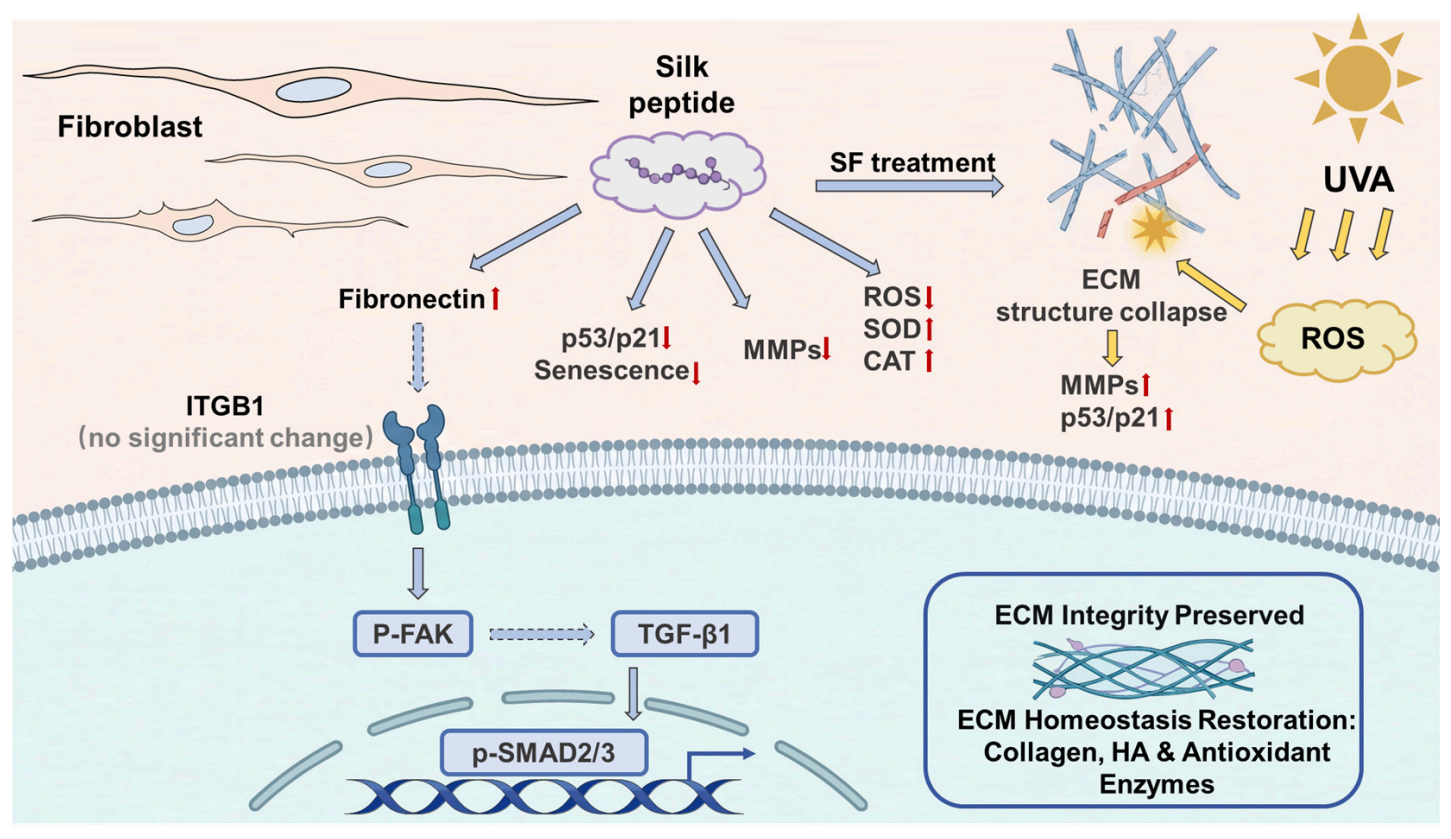
Proposed mechanism of SF against UVA-induced ECM disruption. Solid arrows: direct effects. Dashed arrows: indirect/correlative effects. ITGB1 was not significantly altered; no direct SF–ITGB1 binding is implied.

Collectively, the present findings suggest that SF exerts anti-photoaging effects through a three-tiered mechanism comprising antioxidant defense restoration, senescence suppression, and ECM homeostasis recovery (Figure 7). Excessive ROS generation induced by UVA irradiation constitutes the central initiating event that triggers the photoaging cascade, leading to antioxidant enzyme depletion, cellular senescence activation, and ECM structural disintegration [33]. SF first reconstitutes the oxidative defense system by scavenging excess ROS and restoring SOD and CAT activities. Subsequently, it suppresses the p53-p21 axis to block cellular senescence progression. At the ECM regulatory level, SF is associated with recovery of TGF-β/Smad anabolic signaling via the ITGB1/FAK mechanosensory pathway, which may promote collagen synthesis and limit MMP-mediated proteolysis, potentially contributing to ECM homeostasis restoration.

Based on these findings, we propose a working model in which SF protects against UVA-induced photoaging through a multi-tiered mechanism. As illustrated in Figure 7, UVA irradiation triggers excessive ROS production, which simultaneously induces cellular senescence via the p53-p21 axis and upregulates MMP-mediated ECM degradation, ultimately leading to ECM structure collapse. Post-irradiation SF treatment exerts direct protective effects by scavenging ROS and restoring SOD and CAT activities, suppressing senescence through downregulation of p53/p21, and inhibiting MMP expression, thereby preserving ECM integrity and restoring ECM components such as fibronectin. This ECM preservation maintains the availability of ECM ligands (e.g., fibronectin) for integrin binding, which may indirectly support the recovery of ITGB1/FAK mechanotransduction and downstream TGF-β/Smad signaling, ultimately contributing to ECM homeostasis restoration. Importantly, the proposed connection between SF and integrin signaling is based on correlative observations and indirect evidence; direct binding of SF peptides to integrin receptors remains to be established in future studies.

## 3. Materials and Methods

### 3.1. Materials

Silk fibroin peptide (SF) was obtained from Zhejiang Daldrem Biotechnology Co., Ltd. (Ningbo, China). SF was characterized for molecular weight distribution, peptide size, amino acid composition, purity, and batch identity (Table S1, Supplementary Materials). Human dermal fibroblasts (HDFs) were sourced from Beina Biology (Zhengzhou, China). Key instruments included a confocal microscope (Leica TCS SP8, Leica, Wetzlar, Germany), a microplate reader (Thermo Varioskan ALF, Thermo Fisher Scientific, Waltham, MA, USA), an inverted fluorescence microscope (Olympus IX73, Olympus Corporation, Tokyo, Japan), and a UV crosslinker (SCIENTZ03 PRO, Ningbo Scientz, Ningbo, China).

### 3.2. Cell Culture and UVA Photoaging Model

#### 3.2.1. Cell Culture

HDFs were cultured at 37 °C in a humidified 5% CO_2_ atmosphere using Dulbecco’s Modified Eagle Medium (DMEM; HyClone, Logan, UT, USA) supplemented with 10% FBS and 1% penicillin-streptomycin (Gibco, Frederick, MD, USA). Cells at passages ≤20 were used for all experiments.

For photoaging induction, HDFs seeded at 80–90% confluence were washed twice with PBS and exposed to UVA (365 nm) at doses of 0–30 J/cm^2^ in a thin PBS layer. PBS was replaced with serum-free DMEM with or without SF treatment as detailed in Section 3.2.2, and cells were incubated for 24 h prior to analysis.

#### 3.2.2. SF Treatment

SF was dissolved in serum-free DMEM to final concentrations of 12.5, 50, and 100 μg/mL immediately before use. SF was not present during UVA irradiation; immediately after UVA exposure, PBS was replaced with serum-free DMEM containing SF at the indicated concentrations (or vehicle for controls), and cells were incubated for 24 h prior to analysis.

### 3.3. Phenotype Assessment

Cytotoxicity (CCK-8): HDFs seeded in 96-well plates (5 × 10^4^ cells/well) were treated with SF (0–1000 μg/mL) for 24 h. After adding 10 μL CCK-8 solution (Meilun Biotechnology, Dalian, Liaoning, China) per well and incubating for 1 h at 37 °C in the dark, absorbance was read at 450 nm. Viability was calculated as (A_sample/A_control) × 100%.

Senescence (SA-β-gal): Cellular senescence was detected using a SA-β-gal staining kit (Beyotime, Shanghai, China). Following UVA irradiation and 24 h SF treatment, cells were fixed and stained overnight at 37 °C. Blue-positive cells were counted in five random fields per well.

ROS measurement: Intracellular ROS was measured using the DCFH-DA probe. After 24 h SF treatment, harvested cells were incubated with 10 μM DCFH-DA for 30 min at 37 °C in the dark, and fluorescence was quantified (Ex/Em = 488/525 nm).

Sample preparation: Post-treatment, culture supernatants were collected and centrifuged. Adherent cells were lysed on ice in RIPA buffer containing PMSF (Beyotime). Both supernatants and lysates were stored for subsequent assays.

Enzyme activities and ELISA: SOD and CAT activities in lysates were determined using commercial kits (Beyotime). Protein levels of Col I, Col III, and elastin in lysates, as well as MMP-1 and HA in supernatants, were quantified using ELISA kits (Absin Bioscience, Shanghai, China). All procedures followed the manufacturers’ protocols, with concentrations calculated from standard curves using absorbance at 450 nm.

### 3.4. Immunofluorescence Staining

HDFs grown on glass-bottom dishes were fixed with 4% PFA after 24 h SF treatment. After blocking, cells were incubated overnight at 4 °C with primary antibodies against Col I, elastin, or MMP-1 (Proteintech, Wuhan, Hubei, China, 1:1000), followed by Alexa Fluor 594-conjugated secondary antibody (1:700) at room temperature. Nuclei were counterstained with DAPI. Images were acquired via confocal microscopy (Leica TCS SP8). For visualization of global ECM fibril networks (Col I, elastin, MMP-1 staining), a 20×/0.8 NA objective (200× total magnification, scale bar = 200 μm) was used; this magnification is standard for dermal ECM research to capture overall network continuity across multiple cell populations, as high-magnification fields cannot represent the global ECM structure phenotype.

### 3.5. qRT-PCR Analysis

Total RNA was extracted using an RNA Extraction Kit (Servicebio, Wuhan, China). RNA purity and concentration were determined with a Nanodrop 2000 spectrophotometer (Thermo Fisher Scientific, Dreieich, Germany). For cDNA synthesis, equal amounts of RNA were reverse-transcribed using a Servicebio kit on a thermal cycler (Eastwin Life Sciences, Beijing, China). Quantitative PCR was run on a Bio-Rad CFX96 system with SYBR Green master mix(Bio-Rad Laboratories, Hercules, CA, USA). The cycling protocol was: 95 °C for 30 s, then 40 cycles of 95 °C for 15 s and 60 °C for 30 s. Primer sequences are listed in Table 1.

### 3.6. Western Blot Analysis

Total protein extracted in RIPA buffer was resolved by SDS-PAGE, transferred to PVDF membranes, and blocked with 5% non-fat milk for 2 h. Membranes were probed overnight at 4 °C with primary antibodies against Smad2, p-Smad2, Smad3, p-Smad3, Collagen I, TGF-β1, α-SMA, Fibronectin, ITGB1, FAK, p-FAK, and β-actin (Servicebio, Wuhan, Hubei, China/Proteintech, Wuhan, Hubei, China/Affinity, Changzhou, Jiangsu, China, 1:1000–1:3000), followed by HRP-conjugated secondary antibodies. Signals were visualized via enhanced chemiluminescence and densitometrically quantified using ImageJ (version 1.54d;Java 1.8.0_345, 64-bit).

### 3.7. Statistical Analysis

Data are expressed as mean ± SD. Group comparisons were assessed by one-way ANOVA followed by Tukey’s test (GraphPad Prism 10.3.1), with * *p*< 0.05 considered statistically significant.

## 4. Conclusions

This study provides evidence that SF may contribute to ECM homeostasis restoration in UVA-induced photoaging, with its effects potentially involving the ITGB1/FAK–TGF-β/Smad signaling axis. Mechanistically, SF dose-dependently attenuates oxidative stress and cellular senescence, reverses UVA-induced downregulation of *COL1A1*, *COL3A1*, and *ELN*, and suppresses the expression of *MMP1*, *MMP3*, and *MMP9*. At the molecular level, SF treatment was associated with preservation of fibronectin, an ECM ligand for ITGB1, and enhanced FAK phosphorylation, whereas ITGB1 expression itself was not significantly altered. This suggests that the observed recovery of integrin-mediated signaling may occur primarily through ECM ligand preservation rather than direct receptor upregulation, which may indirectly support integrin-mediated mechanotransduction and downstream TGF-β1/Smad2/3-driven anabolic signaling. The recovery of mechanotransduction signaling appears to be closely associated with SF-mediated ROS scavenging and MMP inhibition, which help preserve ECM integrity and ligand availability for integrin binding, rather than reflecting direct SF-integrin interactions. Unlike prior investigations [34] focusing primarily on the antioxidant or MMP-inhibitory properties of silk peptides, the present work suggests that SF rebalances ECM turnover with both activation of matrix synthesis and suppression of proteolytic degradation; these effects are potentially mediated through the ITGB1/FAK–TGF-β/Smad cascade. These findings suggest that SF may act through mechanotransduction-related pathways to mitigate UVA-induced ECM disruption, offering a molecular rationale for further exploration of SF in anti-photoaging strategies targeting ECM homeostasis restoration.

## Figures and Tables

**Table 1:** Primer sequence.

Gene	Primer (5′→3′)	GenBank Accession No.
*GAPDH*	*F:TGAAGGTCGGAGTCAACGGA*	NM_002046.7
	*R:CCATGTAGTTGAGGTCAATGAAGG*	
*COL1A1*	*F:GAACATGACCAAAAACCAAAAGTG*	NM_000088.4
	*R:GGTGTGGAGAAAGGAGCAGAAA*	
*COL3A1*	*F:GCCTCATTAGTCCTGATGGTTCT*	NM_000090.4
	*R:CCGTGGAACATTCAAAGGATT*	
*ELN*	*F:TGGTTTTATTGTTGTGGTTCATTG*	NM_000501.4
	*R:AAAAGGTGTGTTTCATCCAGAGTTAT*	
*MMP1*	*F:GGGGCTTTGATGTACCCTAGC*	NM_002421.4
	*R:TGTCACACGCTTTTGGGGTTT*	
*MMP3*	*F:TTGGGTTCTTTTATTTCTTTACTGG*	NM_002422.5
	*R:ATTGTGCCTTCTACATATCTCTTTCA*	
*MMP9*	*F:TACAGTTTGTTCCTCGTGGCG*	NM_004994.3
	*R:TTCAGGGCGAGGACCATAGAG*	
*Fibronectin1*	*F:GTTATGGAGGAAGCCGAGGTT*	NM_002026.4
	*R:CGGTTTGCGATGGTACAGC*	
*P21*	*F:GCCCGTGAGCGATGGAA*	NM_000389.5
	*R:GCAGCAGAGCAGGTGAGGTG*	
*P53*	*F:GCTGCTCAGATAGCGATGGTC*	NM_000546.6
	*R:CATGTAGTTGTAGTGGATGGTGGTAC*	

## Data Availability

The original contributions presented in this study are included in the article/Supplementary Material. Further inquiries can be directed to the corresponding authors.

## Supplementary Materials

The following supporting information can be downloaded at https://www.mdpi.com/article/10.3390/molecules31162820/s1: Figure S1: Cytotoxicity of SF on HDFs; Figure S2: UVA dose-dependent cell viability in HDFs; Table S1: Physicochemical properties of silk fibroin peptide (SF).

## Abbreviations

The following abbreviations (listed alphabetically) are used in this manuscript:

ANOVA	Analysis of variance
CAT	Catalase
DAPI	4′,6-Diamidino-2-phenylindole
DCFH-DA	2′,7′-Dichlorodihydrofluorescein diacetate
DMEM	Dulbecco’s modified Eagle medium
ECM	Extracellular matrix
ELISA	Enzyme-linked immunosorbent assay
FAK	Focal adhesion kinase
FBS	Fetal bovine serum
HDFs	Human dermal fibroblasts
HRP	Horseradish peroxidase
ITGB1	Integrin beta 1
MMP	Matrix metalloproteinase
PBS	Phosphate-buffered saline
PVDF	Polyvinylidene difluoride
qRT-PCR	Quantitative real-time polymerase chain reaction
ROS	Reactive oxygen species
SA-β-gal	Senescence-associated β-galactosidase
SD	Standard deviation
SDS-PAGE	Sodium dodecyl sulfate–polyacrylamide gel electrophoresis
SF	Silk fibroin peptide
SOD	Superoxide dismutase
TGF-β	Transforming growth factor-beta
UVA	Ultraviolet A
