# Supplementary material for: Silk Fibroin Peptides Promote Extracellular Matrix Homeostasis in Photoaging via Modulation of the ITGB1/FAK-TGF-β/Smad Signaling Axis
Source: Molecules. 2026 Aug 13;31(16):2820. doi: 10.3390/molecules31162820 (PMC13515133; doi:10.3390/molecules31162820)
Supplement: Supplementary file 1 [file molecules-31-02820-s001.zip › molecules-4421869-supplementary.pdf]

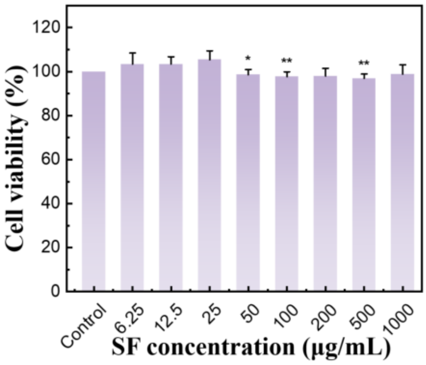

Figure S1. Cytotoxicity of SF on HDFs.

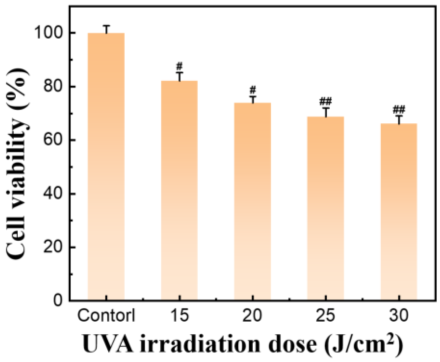

Figure S2. UVA dose-dependent cell viability in HDFs.

Table S1. Physicochemical properties of silk fibroin peptide (SF).

Parameter	Description
Source	Zhejiang Dade Meimei Biotechnology Co., Ltd. (Zhejiang, China)
Batch number	20250301
Appearance	Light yellow powder, characteristic odor, no visible foreign matter
Molecular weight distribution	97.04% of hydrolysates with Mw <1000 Da; relative percentage in 189–10000 Da range: 44.32%
Peptide size range	Mainly 2–6 amino acids (major peaks at ~180 and 245 Da, corresponding to 2–4 residues)
Amino acid composition	Glycine, alanine, serine, and tyrosine collectively account for >80% of total amino acids; the remaining includes aspartic acid, glutamic acid, hydroxyproline, arginine, threonine, proline, valine, methionine, cystine (Cys-Cys), isoleucine, leucine, histidine, phenylalanine, and lysine
Purity	Protein content (dry basis): 93.8%; oligopeptide content (dry basis): 64.38%
Solubility	Soluble in water
Endotoxin level	< 0.5 EU/mg
Storage conditions	store at 4 °C
